# Comorbid Depression in Hospitalised Youth With Anorexia Nervosa: Characteristics, Correlates, and Impact on Weight Gain and Treatment Duration

**DOI:** 10.1002/erv.70130

**Published:** 2026-05-27

**Authors:** Antonia Wiese, Sabine Arnold, Sarah Zaid, Antonia E. Müller, Christoph U. Correll, Charlotte Jaite

**Affiliations:** ^1^ Department of Child and Adolescent Psychiatry, Psychosomatic Medicine and Psychotherapy Charité – Universitätsmedizin Berlin Corporate Member of Freie Universität Berlin, Humboldt Universität zu Berlin, and Berlin Institute of Health Berlin Germany; ^2^ Department of Psychology IB Hochschule für Gesundheit und Soziales Berlin Germany; ^3^ Department of Psychology University of Potsdam Potsdam Germany; ^4^ Department of Psychiatry and Molecular Medicine Zucker School of Medicine at Hofstra/Northwell Hempstead New York USA; ^5^ Center for Psychiatric Neuroscience Feinstein Institute for Medical Research Manhasset New York USA; ^6^ German Center for Mental Health (DZPG), partner site Berlin Berlin Germany; ^7^ German Center for Child and Adolescent Health (DZKJ), partner site Berlin Berlin Germany; ^8^ Einstein Center for Population Diversity (ECPD) Berlin Germany; ^9^ Department of Clinical Psychology and Psychotherapy in Childhood and Adolescence University of Hildesheim Hildesheim Germany

**Keywords:** anorexia nervosa, depression, inpatients, psychotropic medication, youth

## Abstract

**Objective:**

Anorexia nervosa (AN) is associated with high rates of comorbidities. The impact of comorbid depression‐spectrum disorders (DSDs) in AN patients on the outcomes of inpatient treatment is insufficiently studied.

**Method:**

Retrospective study of youth with AN (9–18 years), admission weight < 10th body mass index (BMI) percentile, consecutively hospitalised in a child and adolescent psychiatry university department. Patients with AN+DSD as the only psychiatric comorbidity were compared with AN patients without psychiatric comorbidity (ANnoComorbidity).

**Results:**

Among 220 female AN inpatients (median = 15.0 years) 62 had AN+DSD versus 158 had ANnoComorbidity. Patients with AN+DSD were more likely to report childhood emotional abuse (*p* = 0.020), and to receive psychotropic medications (any: *p* < 0.001; antidepressants: *p* < 0.001; antipsychotics: *p* = 0.001). Moreover, AN+DSD had less average weekly weight gain (*p* = 0.012) and longer inpatient treatment (*p* < 0.001). Independent correlates of less average weekly weight gain included lower admission BMI *z*‐score (*p* < 0.001), antipsychotic (*p* = 0.002) and antidepressant (*p* = 0.004) treatment (*R* ^2^ = 0.17, *p* < 0.001). Independent correlates of longer hospitalisation included lower admission BMI *z*‐scores (*p* < 0.001), antipsychotic (*p* < 0.001) or antidepressant (*p* < 0.001) treatment (*R* ^2^ = 0.27, *p* < 0.001).

**Conclusion:**

AN+DSD were hospitalised longer and gained less average weight/week compared to ANnoComorbidity. Lower admission weight, antipsychotic or antidepressant co‐treatment were independently associated with less average weight gain and longer inpatient stays.

## Highlights

- Patients with anorexia nervosa and depression‐spectrum disorder had less average weekly weight gain versus those without any psychiatric comorbidities.
- Patients with anorexia nervosa and depression‐spectrum disorder had a longer inpatient treatment versus those without any psychiatric comorbidities.
- Patients with anorexia nervosa and depression‐spectrum disorder received inpatient psychotropic medications more often versus those without any psychiatric comorbidities, expectedly a higher prescription of antidepressants, but also of antipsychotics.

## 1 Introduction

Anorexia nervosa (AN) is the third most common disorder (after asthma and obesity) in (female) children and adolescents (Gonzalez et al. 2007; Mathews et al. 2011) and is characterised by underweight, body image disorder, pathological eating behaviour and strong fear of weight gain (Dilling and Freyberger 2019; Hebebrand et al. 2024; Herpertz‐Dahlmann 2015), as well as poor outcomes, including chronicity, mortality and potential years of life lost (Chan et al. 2023; Solmi et al. 2024). Moreover, more than 50% of those affected by AN also report comorbid affective disorders (Bühren et al. 2014; Herpertz‐Dahlmann 2015; Salbach‐Andrae et al. 2008). Comorbid depression and depressive personality traits are considered risk factors for AN and contribute to an unfavourable course of the disorder (Marzola et al. 2020; Steinhausen 2002). Patients with eating disorders exhibit a similar severity of depressive symptoms as patients with depression disorders only (Voderholzer et al. 2019). Moreover, mood is significantly influenced by starvation/underweight in patients with AN, and depressive symptoms in turn affect eating behaviour (Eskild‐Jensen et al. 2020; Herpertz‐Dahlmann 2015). This relationship may explain the severity of AN and comorbid depression. However, no final conclusion about causal relationships can be made according to the current state of research (Herpertz‐Dahlmann 2015). Weight rehabilitation is one of the most important therapeutic goals during inpatient treatment of patients with AN and is influenced by admission weight, age at AN illness onset, age at admission, duration of AN and motivation to change the anorectic eating behaviour (Collin et al. 2010; Deutsche Gesellschaft für Psychosomatische Medizin und Ärztliche Psychotherapie (DGPM) 2019; Hillen et al. 2015; National Institute for Health and Care Excellence (NICE) 2020).

In their systematic review, Eskild‐Jensen et al. (2020) identified studies that examined an impact of comorbid depression on weight gain during treatment in adolescents and adults with AN (inpatient, partial hospital and outpatient setting). The included studies present a very heterogeneous pattern in setting, research objective and findings (Meule et al. 2020). As shown in the study by Berona et al. (2018; *n* = 102 patients; 16.4 ± 2.9 years; partially hospitalised adolescent and adult sample), patients with AN and lower weight gain were more likely to report an affective disorder than those patients with medium and rapid weight gain. However, it should be noted that patients with AN and rapid weight gain had a lower BMI at admission compared to patients in the low weight gain group (higher BMI at admission). This fact could also explain the described effect of lower weight gain (Meule et al. 2023). In outpatients with AN, lower weight gain was also associated with comorbid depression (Marcoulides and Waller 2012; *n* = 32 female outpatients; 26.1 ± 8.7 years). Panero et al. (2021; *n* = 87 female inpatients; 20.6 ± 4.2 years) showed that patients with AN and comorbid depression had lower weight gain and caloric intake compared to patients with AN and without comorbid depression. In this study, patients with AN and comorbid depression had a higher BMI at admission (this difference was not significant), which may also have influenced the results of lower weight gain in this group (Meule et al. 2023). However, Meule et al. (2023) reported that the results of Panero et al. (2021) and Berona et al. (2018) would not hold after applying statistical corrections. In contrast, the study by Meule et al. (2023; *n* = 3011 inpatients; 24.3 ± 10.5 years) indicates that the presence of severe depressive symptoms is associated with a higher BMI at discharge. However, this effect can be explained by a longer duration of treatment. It is conceivable that comorbid depression could act as a contributing factor, possibly because longer inpatient treatment contributes positively to long‐term recovery (Schlegl et al. 2014; Zeeck et al. 2005). Schlegl et al. (2014; *n* = 435 female inpatients; 26.36 ± 9.02 years) showed that patients with AN and no change or even worsening of eating disorder psychopathology at discharge had more depressive symptoms compared to patients with significant change in eating disorder psychopathology during hospitalisation. Similar results were obtained by Schlegl et al. (2016; *n* = 238 female inpatients; 15.7 ± 1.1 years) showing that both longer inpatient stay and severe comorbid depression were associated with serious eating disorder psychopathology change in adolescents during inpatient treatment.

In summary, the current evidence of inpatient weight change in youth with AN and comorbid depression‐spectrum disorders is scarce. Existing studies mostly had low to medium sample sizes, focused predominantly on outpatient settings and adult patients, making the results only partially transferable to children and adolescents. There is a lack of studies focussing on inpatient weight gain progression with regard to the severity of AN as well as the presence of comorbid depression‐spectrum disorders. Existing studies considered comorbid depressive disorders in patients with AN mostly as a co‐variable (Eskild‐Jensen et al. 2020). Given the high prevalence of psychiatric comorbidities, especially of depression, in patients with AN, it is important to examine the impact of comorbid depression on weight gain during inpatient treatment (Blinder et al. 2006; Herzog et al. 1992; Marcoulides and Waller 2012). Identifying specific subgroups of patients with AN enables the development of treatment concepts that are more adapted to these groups and, thus, could have a favourable impact on prognosis.

Therefore, the first aim of the present study was to investigate differences in weight‐related parameters at hospital admission and discharge as well as changes in weight‐related parameters during inpatient treatment between children and adolescents with AN and a depression‐spectrum disorder (AN+DSD) versus those without any psychiatric comorbidities (ANnoComorbidity). The second aim was to investigate the clinical characteristics and independent correlates of AN+DSD versus ANnoComorbidity.

The following hypotheses were tested: inpatients with AN+DSD compared to ANnoComorbidity (1) do not differ in BMI *z*‐score at admission, (2) but have lower BMI *z*‐score at discharge, (3) lower average weekly weight gain (in kg) during inpatient treatment, (4) higher rates of psychiatric medication use, and (5) longer duration of inpatient treatment.

## 2 Method

### 2.1 Design, Setting, Patients

This chart review was performed as a part of a larger retrospective cohort study at the department of child and adolescent psychiatry Charité Berlin, Germany (Arnold et al. 2022; Arnold et al. 2023; Frank et al. 2023) in which patients treated as inpatients from January 1990 to June 2015 for AN were included. Inclusion criteria of the present analysis were female sex, age ≤ 18 years at admission, body mass index (BMI) percentiles at admission < 10, a diagnosis of AN restrictive type (AN‐R), AN binge‐purge type (AN‐BP), or atypical AN according to the International Classification of Diseases (World Health Organization [WHO] 1992), absence of menstruation (without taking the contraceptive pill in patients with AN‐R and AN‐BP) and inpatient treatment duration ≥ 14 days. Furthermore, patients from 1994 onwards were included, as this was the year in which ICD‐10 came into force. An upper age limit of 18 years was applied, reflecting the legal definition of adulthood in Germany. The lower age limit was defined by the age of the youngest patient included in the analysis (9 years). For the subdivision of the two patient groups, inpatients with comorbid depression‐spectrum disorders as the only psychiatric comorbidity and inpatients without any psychiatric disorders were considered. In case of multiple inpatient stays at the Charité University Hospital child and adolescent psychiatry, only the first admission was taken into account to ensure homogeneity of the study sample. All patients underwent a CBT‐based eating disorder‐specific multimodal inpatient treatment programme with nutritional counselling, body therapy, individual and group psychotherapy as well as family sessions.

### 2.2 Study Procedures and Measures

Data relevant to the study were retrieved from patient records using a standardised template and supplemented with information of the departmental baseline documentation database. The existing variables in the basic documentation system were supplemented by chart review‐extracted information on eating disorder‐specific and weight‐related variables as well as psychopharmacological treatment. The basic documentation system included sociodemographic and anamnestic data, psychopathological information (e.g., eating disorder, comorbidities) and somatic findings (e.g., weight at admission/discharge), and the diagnostic assessment according to the ‘Multiaxial classification of child and adolescent psychiatric disorders’ (Englert and Poustka 1995). For the present study, the following clinical parameters were considered: sex, intelligence (clinical assessment according to the ICD‐10 IQ‐categories very high > 129, high = 115–129, average = 85–114, below average = 70–84, mental retardation = < 70), ICD‐10 (atypical) diagnoses of AN (AN‐R = F50.00, AN‐BP = F50.01, atypical AN = F50.1) and affective disorders (yes/no; mild depressive episode = F32.0; moderate depressive episode = F32.1; severe depressive episode without psychotic symptoms = F32.2; recurrent depressive disorder, current episode mild = F33.0; recurrent depressive disorder, current episode moderate = F33.1; recurrent depressive disorder, current episode severe without psychotic symptoms = F33.2; dysthymia = F34.1; other persistent mood [affective] disorders = F34.8), age of AN onset, duration of AN, age at admission, year of admission, weight loss before admission (weight at admission compared to premorbid body weight), weight‐related parameters at hospital admission and discharge, history of childhood abuse (yes/no; types: physical/emotional/sexual), psychotropic medications during inpatient treatment (yes/no; types: antipsychotics/antidepressants/anxiolytics/other types of psychotropic medication/psychostimulants; average number), and inpatient treatment duration.

Based on weight in kilogrammes (kg) and height in metres (m) data, BMI‐related values were calculated using a specific BMI calculator (Pedz): BMI (kg body weight/metres body height^2^), BMI percentiles (age‐ and sex‐adjusted BMI; Kromeyer‐Hauschild et al. 2001), BMI *z*‐scores (BMI‐M/SD reference population: *n* = 34.422 girls and boys between 0 and 18 years) and kg < 1. BMI percentile. To calculate weight gain, the admission weight was subtracted from the discharge weight. Average weekly weight gain was calculated by dividing the total weight gain by the duration of inpatient treatment.

Patient data registered and stored during hospital treatment were anonymised for research aims before data entry and evaluation. The Ethics Committee of the Charité – Universitätsmedizin Berlin approved this retrospective chart review granting a waiver of informed consent.

### 2.3 Statistical Analysis

Statistical analyses were performed using IBM Statistical Package for the Social Sciences (SPSS Version 27; IBM Corp 2020). The significance level was set at *p* ≤ 0.05, and all effects were tested for significance in a hypothesis‐driven undirected manner.

To calculate possible differences in the characteristics and weight‐related data of the two examined patient groups (AN + DSD, ANnoComorbidity), a two‐sided t‐test for independent samples for interval‐scaled variables or *X* ^*2*^‐tests for nominal data were used. For interval‐scaled variables, the normal distribution was reviewed using the Kolmogorov‐Smirnov and Shapiro‐Wilk tests. If the normal distribution assumption was violated, a Mann‐Whitney U test was performed instead of the t‐test. Whenever a cell size was < 5, Fisher's exact test was used.

To detect independent correlates of average weekly weight change and inpatient treatment duration, multi‐variable stepwise backward elimination regression analyses were performed. Bivariate correlation analyses of sample characteristics, weight‐related data, psychotropic medications, and corresponding dependent variables (average weekly weight change, inpatient treatment duration) were performed to preselect variables. Only variables that showed a significant correlation with the dependent variable were included in the regression model (see Supporting Information S1: Appendix 1 and 2 for more information about the variable selection). If the normal distribution of the residuals was violated, the empirical distribution function of the residuals in a bootstrapping procedure instead of assuming normal distribution was used (Schlittgen 2012). Therefore, the standard errors and, thus, the *p*‐values of the final model were estimated from the stepwise regression using bootstrapping. To prevent multicollinearity (*VIF* = 5–10 can be problematic; *VIF* > 10 = strong multicollinearity; *tolerance* < 0.1 indicate serious problems), variables were excluded if strong intercorrelations (*r* > 0.80) were present, if they yielded similar results, or if they could be derived from other variables in the dataset (Field 2009).

The effect sizes were interpreted according to Cohen (1988). For a two‐sided t‐test for independent samples, *d* = 0.20 is considered a small effect, *d* = 0.50 a medium effect, and *d* = 0.80 a large effect. For the *X* ^*2*^‐test, Cramér's *V* = 0.10 is considered a small effect, *V* = 0.30 a medium effect, and *V* = 0.50 a large effect. For multiple regression, Cohen's *f* ^2^ = 0.02 is interpreted as a weak effect, *f* ^2^ = 0.15 as a medium effect, and *f* ^2^ = 0.35 as a strong effect.

## 3 Results

### 3.1 Sample Characteristics

Of the initial 379 patients, 220 were included in the final analyses. A total of 159 patients were excluded from the analyses for the following reasons: male sex (*n* = 10), BMI percentiles at admission ≥ 10 (*n* = 34), length of inpatient treatment < 14 days (*n* = 8), year of admission < 1994 (*n* = 2), additional comorbidities (beside AN+DSD; *n* = 66), and AN‐R and AN‐BP patients who were regularly menstruating at admission (without oral contraceptive; *n* = 39).

**FIGURE 1:**
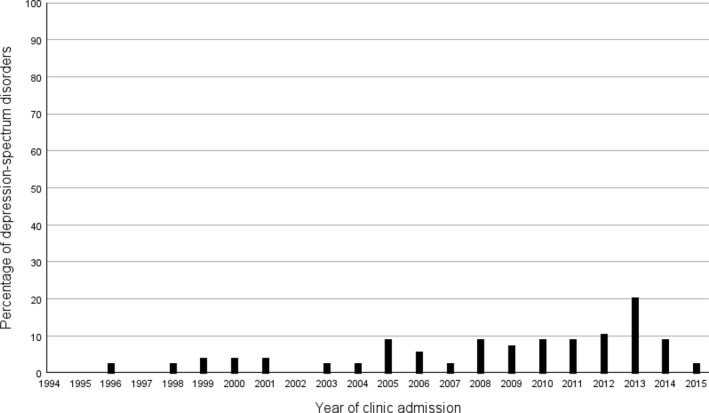
Frequency of comorbid depression diagnoses between 1994 and 2015.

The final sample consisted of 220 patients with an age range of 9–18 years and with a median age of 15.0 years at admission (interquartile range: 13.8, 16.4). Altogether, 62 (28.2%) patients were diagnosed with comorbid depression‐spectrum disorders as their only psychiatric comorbidity (AN+DSD), while 158 patients (71.8%) had no psychiatric comorbidities (ANnoComorbidity). There were no significant differences between the patient groups concerning the age at AN onset (*p* = 0.916), age at admission (*p* = 0.932) and duration of AN (*p* = 0.881). Patients with AN+DSD were admitted significantly more often in later years compared to patients with ANnoComorbidity (year of admission). For more details on the time course, please refer to Figure 1. Weight loss before hospital admission (*p* = 0.987) and all anthropometric measures at admission did not differ significantly between the groups (*p* = 0.115–0.312). However, the patient groups differed significantly in the prevalence of childhood emotional abuse (*p* = 0.020; AN+DSD = 8.1% vs. ANnoComorbidity = 1.3%).

### 3.2 Inpatient Treatment

**FIGURE 2:**
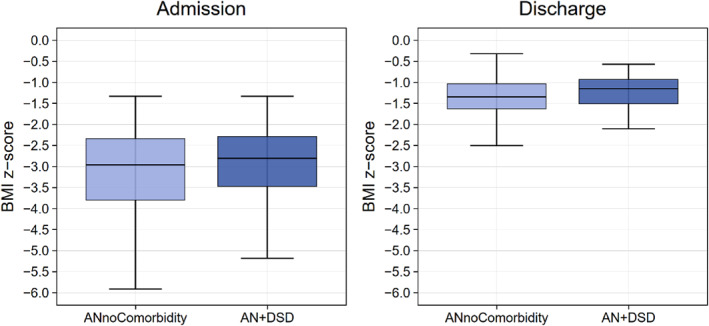
Weight‐related group comparisons at admission and at discharge. Each boxplot displays the median (horizontal line within the box), the interquartile range (IQR; box boundaries represent the 25th (Q1) and 75th (Q3) percentiles), and the whiskers indicating the range of values within IQR × 1.5.

**FIGURE 3:**
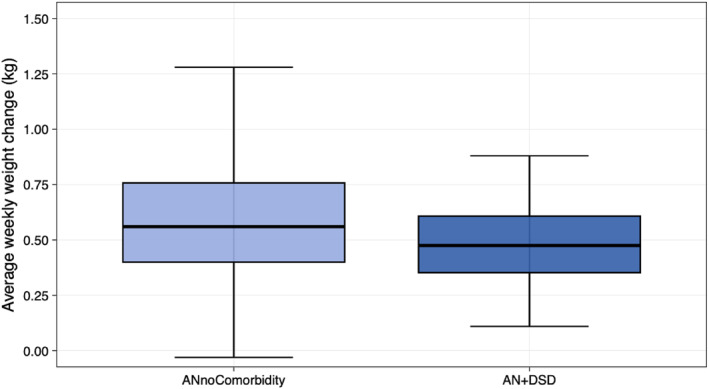
Group comparison of average weekly weight change during inpatient treatment. Each boxplot displays the median (horizontal line within the box), the interquartile range (IQR; box boundaries represent the 25th (Q1) and 75th (Q3) percentiles), and the whiskers indicating the range of values within IQR × 1.5.

**FIGURE 4:**
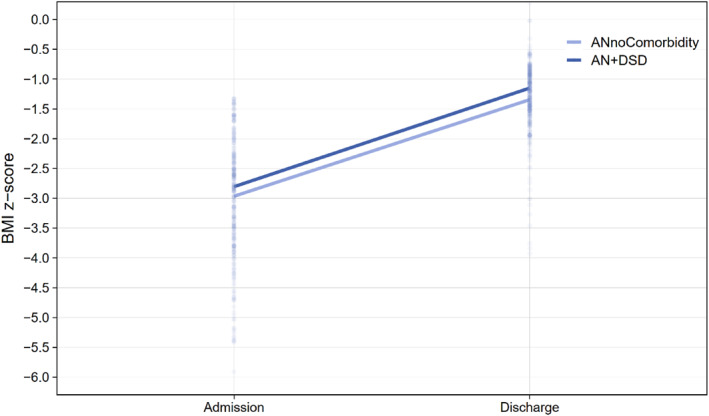
Weight‐related group comparisons from admission to discharge. Median BMI *z*‐scores at admission and at discharge. The lines connect the respective median values across time points. Individual data points are displayed in the background.

The groups differed significantly in terms of duration of inpatient treatment (*p* < 0.001; AN+DSD > ANnoComorbidity) and average weekly sex‐ and age‐adjusted weight gain (kg: *p* = 0.013; AN+DSD < ANnoComorbidity). There were no significant differences in weight‐related parameters at discharge (BMI *z*‐score: *p* = 0.069). Compared to patients with ANnoComorbidity, patients with AN+DSD more often received psychotropic medications during hospitalisation, both globally (any: *p* < 0.001; ANnoComorbidity = 10.8%, AN+DSD = 50.0%), and related to specific medications, that is, antidepressants (*p* < 0.001; AN+DSD = 35.5%, ANnoComorbidity = 3.8%) and antipsychotics (*p* < 0.001; AN+DSD = 25.8%, ANnoComorbidity = 8.2%) (see Table 1, Figure 2, 3, 4).

**TABLE 1:** Clinical characteristics at admission and intra‐treatment of inpatient youth with anorexia nervosa with comorbid depression‐spectrum disorders versus without psychiatric comorbidities.

	Total (*N* = 220)		AN without psychiatric comorbidities (*n* = 158)		AN + depression‐spectrum disorders (*n* = 62)		t‐test χ^2^‐test Mann‐Whitney U test			Effect sizes
							*df*	*t* *χ* ^2^ *U*	*p*	*d* *V* *r*
Female	220	(100.0)	160	(94.7)	62	(98.4)	—	—	—	—
Affective disorders, at least one (ICD‐10, *n (%))*										
Depressive episode	36	(16.4)	0	(0.0)	36	(58.1)	—	—	—	—
Persistent mood [affective] disorder	30	(13.6)	0	(0.0)	30	(48.4)	—	—	—	—
Recurrent depressive disorder	1	(0.5)	0	(0.0)	1	(1.6)	—	—	—	—
Age at onset, years (Mdn (Q1, Q3))	14.0	(12.9, 15.2)	14.0	(12.8, 15.2)	14.0	(13.0, 15.0)	—	4911.50	0.916	0.01
Duration of AN, months (M ± SD)	10.4	± 8.4	10.5	± 9.0	10.3	± 6.9	217	0.15	0.881	0.02
Age at admission, years (Mdn (Q1, Q3))	15.0	(13.8, 16.4)	14.9	(13.8, 16.5)	15.1	(13.8, 16.4)	—	4862.00	0.932	−0.01
Year of admission (Mdn (Q1, Q3))	2006.0	(2001.0, 2011.0)	2005.0	(2000.0, 2009.0)	2010.0	(2005.8, 2013.0)	—	7000.50	**<** 0.001*	0.33
Primary amenorrhoea (*n* (%))	46	(20.9)	37	(23.4)	9	(14.5)	1	1.66	0.198	0.09
Secondary amenorrhoea (*n* (%))	153	(69.5)	107	(67.7)	46	(74.2)	1	0.58	0.446	0.05
Oligomenorrhoea (*n* (%))	3	(1.4)	3	(2.0)	0	(0.0)	—	—	—	—
Eating disorders (ICD‐10, *n* (%))							2	4.75	0.093	0.15
AN‐R	165	(75.0)	122	(77.2)	43	(69.4)	—	—	—	—
AN‐BP	35	(15.9)	20	(12.7)	15	(24.2)	—	—	—	—
Atypical AN	20	(9.1)	16	(10.1)	4	(6.5)	—	—	—	—
Intelligence (*n* (%))							3	1.46	0.691	0.10
Very high	15	(6.8)	9	(5.7)	6	(9.7)	—	—	—	—
High	71	(32.3)	50	(31.65)	21	(33.9)	—	—	—	—
Average	131	(59.5)	97	(61.4)	34	(54.8)	—	—	—	—
Below average	3	(1.4)	2	(1.3)	1	(1.6)	—	—	—	—
Mental retardation	0	(0.0)	0	(0.0)	0	(0.0)	—	—	—	—
History of childhood abuse (*n* (%))										
Physical childhood abuse	6	(2.7)	5	(3.2)	1	(1.6)	—	—	1	0.01
Emotional childhood abuse	7	(3.2)	2	(1.3)	5	(8.1)	—	—	0.020*	0.15
Sexual childhood abuse	1	(0.5)	1	(0.6)	0	(0.0)	—	—	—	—
Weight loss in percentage, weight at admission compared to premorbid body weight (Mdn (Q1, Q3))	24.0	(18.6, 30.4)	24.0	(19.3, 30.4)	25.0	(17.2, 30.4)	—	4523.50	0.987	0.00
Weight‐related parameters at hospital admission										
BMI (M ± SD)	14.4	± 1.3	14.3	± 1.3	14.6	± 1.3	218	−1.58	0.115	−0.24
BMI percentile (Mdn (Q1, Q3))	1.0	(1.0, 1.0)	1.0	(1.0, 1.0)	1.0	(1.0, 2.0)	—	5208.00	0.312	0.07
BMI *z*‐score (Mdn (Q1, Q3))	−2.9	(−3.8, −2.3)	−3.0	(−3.8, −2.3)	−2.8	(−3.5, −2.3)	—	5438.00	0.204	0.09
kg < 1. BMI percentile (Mdn (Q1, Q3))	−1.0	(−3.9, 0.0)	−1.7	(−4.1, 0.0)	−0.9	(−3.1, 0.0)	—	5267.50	0.293	0.07
Duration of inpatient treatment, days (M ± SD)	95.0	± 38.3	89.3	± 34.6	109.6	± 43.5	218	−3.64	**<** 0.001*	−0.55
Average weekly change in weight‐related parameters										
kg (Mdn (Q1, Q3))	0.5	(0.4, 0.7)	0.6	(0.4, 0.8)	0.5	(0.3, 0.6)	—	3845.00	0.013*	−0.17
BMI (Mdn (Q1, Q3))	0.2	(0.1, 0.3)	0.2	(0.2, 0.3)	0.2	(0.1, 0.2)	—	3914.00	0.020*	−0.16
BMI percentile (Mdn (Q1, Q3))	0.7	(0.3, 1.1)	0.6	(0.3, 1.1)	0.7	(0.3, 1.3)	—	5213.50	0.457	0.05
BMI *z*‐score (Mdn (Q1, Q3))	0.1	(0.1, 0.2)	0.1	(0.1, 0.2)	0.1	(0.1, 0.1)	—	3836.50	0.012*	−0.17
Weight‐related parameters at hospital discharge										
BMI (Mdn (Q1, Q3))	17.3	(16.5, 17.9)	17.3	(16.4, 18.0)	17.4	(16.6, 17.9)	—	5510.50	0.149	0.10
BMI percentile (Mdn (Q1, Q3))	10.0	(6.0, 17.0)	9.0	(5.0, 15.5)	13.0	(6.8, 19.0)	—	5748.00	0.045*	0.14
BMI *z*‐score (Mdn (Q1, Q3))	−1.3	(−1.6, −1.0)	−1.3	(−1.6, −1.0)	−1.2	(−1.5, −0.9)	—	5671.50	0.069	0.12
kg < 1. BMI percentile (Mdn (Q1, Q3))	0.0	(0.0, 0.0)	0.0	(0.0, 0.0)	0.0	(0.0, 0.0)	—	5038.50	0.283	0.07
Psychotropic medications										
At least one (*n* (%))	48	(21.8)	17	(10.8)	31	(50.0)	1	40.20	**<** 0.001*	0.43
Average number (Mdn (Q1, Q3))	0.0	(0.0, 0.0)	0.0	(0.0, 0.0)	0.5	(0.0, 1.0)	—	6850.50	**<** 0.001*	0.43
Treatment with (*n* (%))										
Antipsychotics	29	(13.2)	13	(8.2)	16	(25.8)	1	12.02	**<** 0.001*	0.23
Antidepressants	28	(12.7)	6	(3.8)	22	(35.5)	1	40.25	**<** 0.001*	0.43
Anxiolytics	1	(0.5)	0	(0.0)	1	(1.6)	—	—	—	—
Other types of psychotropic medications	1	(0.5)	0	(0.0)	1	(1.6)	—	—	—	—
Psychostimulants	0	(0.0)	0	(0.0)	0	(0.0)	—	—	—	—

*Note:* Intelligence: very high = IQ > 129, high = IQ 115–129, average = IQ 85–114, below average = IQ 70–84, Q1 = 1^st^ quartile, Q3 = 3^rd^ quartile, *p ≤ 0.05; statistical methods (1) categorical data = chi‐square tests, (2) continuous data = t‐test, Mann‐Whitney U test, Cohen's *d*: small = 0.20, medium = 0.50, large = 0.80, Cramér's *V*: small = 0.1, medium = 0.3, large = 0.5, *r*: small = 0.1, medium = 0.3, large = 0.5.

Due to missing values, N varies as follows: Age at onset (AN without comorbidities: *n* = 157), duration of illness (AN without comorbidities: *n* = 157), primary amenorrhoea (AN without comorbidities: *n* = 155, AN + DSD: *n* = 61), secondary amenorrhoea (AN without comorbidities: *n* = 155, AN + DSD: *n* = 61), oligomenorrhoea (AN without comorbidities: *n* = 155, AN + DSD: *n* = 61), weight loss (at baseline compared to premorbid body weight (%), AN without comorbidities: *n* = 151, AN + DSD: *n* = 60), kg < 1. BMI percentile at hospital admission (AN without comorbidities: *n* = 156), kg < 1. BMI percentile at hospital discharge (AN without comorbidities: *n* = 157). Bold values represent the significant results.

Abbreviations: AN = anorexia nervosa, AN‐BP = AN, binge‐purge type, AN‐R = AN, restricting type, DSD = depression‐spectrum disorders, IQR = interquartile range, M = mean, Mdn = median, SD = standard deviation.

### 3.3 Clinical Correlates of Average Weekly Weight Change and Inpatient Treatment Duration in Patients With Anorexia Nervosa

Lower average weekly weight gain during inpatient stay was correlated with comorbid depression‐spectrum disorder (*r* = −0.17, *p* = 0.013) and childhood emotional abuse (*r* = −0.15, *p* = 0.030). The earlier the treatment began in the study period (year of admission), the lower the average weekly weight gain was (*r* = −0.15, *p* = 0.027). Less average weekly weight gain was also correlated with a higher BMI *z*‐score at hospital admission (*r* = −0.40, *p* < 0.001), longer inpatient stay (*r* = −0.39, *p* ≤ 0.001), and treatment with psychotropic medications (*r* = −0.28, *p* < 0.001; antidepressants: *r* = −0.17, *p* = 0.010; antipsychotics: *r* = −0.26, *p* < 0.001). In contrast, larger average weekly weight gain correlated with older age at AN onset (*r* = 0.25, *p* < 0.001) and older age at hospital admission (*r* = 0.27, *p* < 0.001). In addition, larger average weekly weight gain was correlated with a higher BMI at discharge (*r* = 0.32, *p* < 0.001).

Furthermore, longer treatment duration was correlated with comorbid depression‐spectrum disorder (*r* = 0.24, *p* < 0.001), greater weight loss before hospital admission (*r* = 0.20, *p* = 0.004), greater likelihood of receiving psychotropic medications during inpatient treatment (*r* = 0.36, *p* < 0.001; inpatient use of antidepressants: *r* = 0.32, *p* < 0.001 and antipsychotics: *r* = 0.24, *p* < 0.001), a smaller admission BMI *z*‐score (*r* = −0.29, *p* < 0.001) and a less average weekly weight gain during inpatient treatment (kg; *r* = −0.39, *p* < 0.001) (Table 2).

**TABLE 2:** Clinical correlates of average weekly weight change and inpatient treatment duration in youth with anorexia nervosa.

Variable	Average weekly weight change, kg	Duration of inpatient treatment, days
Affective disorders (ICD‐10)	**−0.17** *	**0.24** **
Age at onset, years	**0.25** **	−0.03
Duration of AN, months	0.06	−0.00
Age at admission, years	**0.27** **	−0.04
Year of admission	**−0.15** *	0.04
Primary amenorrhoea	−0.13	0.09
Secondary amenorrhoea	−0.13	0.06
Oligomenorrhoea	0.07	0.02
Intelligence		
Very high	−0.05	0.12
High	−0.05	−0.02
Average	0.10	−0.05
Below average	−0.10	0.02
History of childhood abuse		
Physical childhood abuse	−0.02	−0.00
Emotional childhood abuse	**−0.15** *	−0.05
Sexual childhood abuse	−0.10	0.04
Weight loss in percentage, weight at admission compared to premorbid body weight	0.11	**0.20** **
Weight‐related parameters at hospital admission		
kg	−0.11	**−0.19** **
BMI	**−0.28** **	**−0.27** **
BMI percentile	**−0.40** **	**−0.21** **
BMI *z*‐score	**−0.40** **	**−0.29** **
kg < 1. BMI percentile	**−0.37** **	**−0.23** **
Duration of inpatient treatment, days	**−0.39** **	—
Average weekly change in weight‐related parameters		
kg	—	**−0.39** **
BMI	**0.97** **	**−0.40** **
BMI percentile	**0.31** **	**−0.32** **
BMI *z*‐score	**0.90** **	**−0.30** **
Weight‐related parameters at hospital discharge		
kg	**0.23** **	0.03
BMI	**0.32** **	−0.02
BMI percentile	0.10	−0.07
BMI *z*‐score	0.11	−0.08
kg < 1. BMI percentile	**0.17** *	0.01
Psychotropic medications		
At least one	**−0.28** **	**0.35** **
Average number	**−0.28** **	**0.36** **
Treatment with		
Antipsychotics	**−0.26** **	**0.24** **
Antidepressants	**−0.17** **	**0.32** **
Anxiolytics	−0.11	0.11
Other types of psychotropic medications	−0.11	0.11

*Note:* Bold values represent the significant results.

Abbreviation: AN = Anorexia nervosa.

^*^ *p* ≤ 0.05.

^**^ *p* ≤ 0.01.

### 3.4 Independent Correlates of Clinical Characteristics for Average Weekly Weight Change and Duration of Inpatient Treatment in Anorexia Nervosa

Independent correlates of lower average weekly inpatient weight gain, analysed via multivariable stepwise backward elimination regression analysis, included lower admission BMI *z*‐score (*p* < 0.001), and antipsychotic (*p* = 0.002) and antidepressant (*p* = 0.004) treatment (*R* ^2^ = 0.17, *p* < 0.001) (Table 3). Moreover, independent correlates of longer hospital stay included lower admission BMI *z*‐score (*p* < 0.001), and antipsychotic (*p* < 0.001) and antidepressant (*p* < 0.001) treatment (*R* ^2^ = 0.27, *p* < 0.001) (Table 4).

**TABLE 3:** Multi variable stepwise backward elimination regression analysis of the impact of clinical characteristics on average weekly inpatient weight change in children and adolescents with anorexia nervosa.

						95% Cl. of *B*		Effect size
	*B*	SE	*T*	df	*p*	Lower	Upper	*f* ^2^
Weight at hospital admission, BMI *z*‐score	−0.11	0.02	−5.22	216	**<** **0.001** *	−0.15	−0.07	0.13
Antipsychotics	−0.18	0.05	−2.75	216	**0.002** *	0.26	−0.08	0.04
Antidepressants	−0.13	0.04	−1.96	216	**0.004** *	−0.20	−0.05	0.02

*Note:* Bootstrapping with *n* = 1000, *f* ^2^: small = 0.02, medium = 0.15, large = 0.35. Weight change, kg/week, *R* ^2^ of the overall model: 0.166. Bold values represent the significant results.

Abbreviations: *B* = beta, SE = standard error, CI = confidence interval.

^*^ *p* ≤ 0.05.

**TABLE 4:** Multi stepwise variable backward elimination regression analysis of the impact of clinical characteristics on duration of inpatient treatment in children and adolescents with anorexia nervosa.

						95% Cl. of *B*		Effect size
	*B*	SE	*T*	df	*p*	Lower	Upper	*f* ^2^
Affective disorders (ICD‐10)	10.34	5.81	1.78	215	0.076	−1.11	21.78	0.03
Weight at hospital admission, BMI *z*‐score	−13.00	2.31	−5.63	215	**<** **0.001** *	−17.56	−8.45	0.15
Antipsychotics	23.87	6.97	3.43	215	**<** **0.001** *	10.13	37.61	0.07
Antidepressants	25.66	7.65	3.35	215	**<** **0.001** *	10.58	40.75	0.07

*Note:* *f* ^2^: small = 0.02, medium = 0.15, large = 0.35, *R* ^2^ of the overall model: 0.270. Bold values represent the significant results.

Abbreviations: *B* = beta, CI = confidence interval, SE = standard error.

^*^ *p* ≤ 0.05.

## 4 Discussion

This retrospective chart review of 220 youth inpatients with AN investigated the clinical characteristics and independent correlates of AN+DSD versus ANnoComorbidity. To the best of our knowledge, this is the first study that has examined the isolated influence of comorbid depression‐spectrum disorder on inpatient average weekly weight gain in the treatment of patients with AN. This research gap makes it difficult to compare our results with those of other studies.

Our results indicate that patients with AN+DSD had significantly lower average weekly weight gain and longer inpatient treatment duration. Moreover, patients with AN+DSD received more psychotropic medications (antidepressants, antipsychotics) during hospitalisation compared to ANnoComorbidity patients. In contrast, admission and discharge weights showed no significant between‐group differences.

### 4.1 Characteristics of Inpatient Youth With Anorexia Nervosa With Versus Without Comorbid Depression‐Spectrum Disorders

We did not find significant differences between the AN+DSD and ANnoComorbidity groups in terms of weight‐related parameters at admission and at discharge. This finding is inconsistent with previous studies, which showed that patients with comorbid depression had higher weight at discharge (Meule et al. 2023). This difference in discharge weight compared to other studies might be explained by the length of treatment, since ANnoComorbidity patients spent an average of 89.3 days in the hospital, whereas AN+DSD patients were hospitalised for 109.6 days on average. This interpretation is consistent with Meule et al. (2023), who also assumed that a higher BMI at discharge in the presence of depression is mediated by the duration of inpatient treatment. Moreover, we examined only patients in need of hospital treatment, whereas other studies recruited their patients from different treatment settings, for example, day clinics or outpatient units (Berona et al. 2018; Marcoulides and Waller 2012). Hospitalisation is intended for patients with severe AN and involves more stringent treatment standards, including a target weight for discharge. According to the German S3 guidelines of ‘Diagnosis and treatment of eating disorders’, the weight of children and adolescents should be at the 25th BMI percentile, being at least at the 10th BMI percentile at discharge (Deutsche Gesellschaft für Psychosomatische Medizin und Ärztliche Psychotherapie (DGPM) 2019). This recommendation might also explain the similar weight at discharge in both the AN+DSD and the ANnoComorbidity group.

Our findings that AN+DSD showed a lower average weekly weight gain compared to ANnoComorbidity are in line with previous studies, which showed that a depressive disorder has a negative influence on weight gain/eating disorder psychopathology and is thus considered an unfavourable prognostic factor (Eskild‐Jensen et al. 2020; Panero et al. 2021). However, as stated above, Meule et al. (2023) emphasise that the results of Panero et al. (2021) should be critically examined regarding the analyses. Hence, it can be assumed that depression and AN influence each other (Herpertz‐Dahlmann 2015). According to Eskild‐Jensen et al. (2020), depressive symptoms impact the psychopathology of the eating disorder, for example motivational and biological processes (e.g., appetite), which in turn can impact weight gain. Accordingly, it is possible that biological rather than psychological processes or cognitive processes play a key role in the effects of depression on AN. Given this fact, depressive symptoms should be investigated in future studies in a more differentiated way. One option would be the recording of the individual symptoms/criteria of depression (= psychopathology of depression) in addition to the diagnosis. Furthermore, it should be noted that the effect sizes of our results regarding weekly weight gain were very small, which should be taken into account when interpreting the results. The small effect sizes for weekly weight gain could be due to the severity of eating disorder symptoms per se, as well as due to the additive overlap between the clinical symptoms of AN and depression (e.g., reduced drive, appetite regulation, therapy adherence; Voderholzer et al. 2019), which each can contribute to smaller or slower weight gain. Moreover, patients with comorbid depression had a longer inpatient treatment duration, which could further contribute to the overall greater absolute weight gain.

We analysed data from a survey period of more than 20 years (1994–2015). Our results show that the average weekly weight gain was lower, the earlier the treatment was administered (year of admission). While the treatment programs in the 90s were probably less evidence‐based due to limited research data (with regard to external conditions), they were also likely less demanding in expected weight gain per week, likely due to fear of refeeding syndrome. However, the German S3 guideline (in place since 2010) recommends a weekly inpatient weight gain of 500–1000g and provides recommendations that allow a standardised approach to the treatment of AN patients (Deutsche Gesellschaft für Psychosomatische Medizin und Ärztliche Psychotherapie (DGPM) 2019). There is also a steady growth of empirical studies that expand the knowledge about AN, including that high caloric refeeding is possible and safe (Dalenbrook et al. 2022; Haas et al. 2021). In the case of depressive disorders, it can likewise be assumed that contributors to a more standardised diagnostic approach have included the evidence‐based German S3 guidelines of ‘National Care Guideline for Unipolar Depression’ introduced in 2009, the mandatory use of ICD‐10 coding from 2000 onwards, a changing understanding of mental health (e.g., increasing destigmatisation), and improved access to psychosocial care (Bundesärztekammer (BÄK) et al. 2022). These factors may represent possible reasons for the increase in the diagnostic prevalence of depressive disorders as well as for the greater severity of depressive episodes (Bretschneider et al. 2018; Steffen et al. 2019). Secular changes may also play a role. Studies indicate that the age of menarche has tended to occur earlier since the 1990s (Kahl et al. 2007; McDowell et al. 2007). An earlier pubertal maturation has been associated with an increased risk of depressive symptoms and body dissatisfaction (Mendle et al. 2007; Wertheim et al. 2004). In addition, societal changes such as the rise of social media and idealised beauty standards may further contribute to body dissatisfaction and thereby promote depressive symptoms (Perloff 2014; Tiggemann and Slater 2013). These developments represent relevant limitations, as differences between groups may partly be explained by changes in diagnostic criteria, societal influences, or developmental biological factors.

In our study, we identified lower admission BMI *z*‐score and more frequent administration of psychotropic medications as correlates of less average weekly weight gain and longer hospitalisation. These results can be attributed to those predictors being proxy measures of the severity of AN (Brand‐Gothelf et al. 2014; Schlegl et al. 2016). Accordingly, it is also possible that in addition to treating depressive mood disorders, antidepressant and, especially, antipsychotic medications are also used to treat other symptoms, such as fear of gaining weight and physical hyperactivity (Deutsche Gesellschaft für Psychosomatische Medizin und Ärztliche Psychotherapie (DGPM) 2019; Frank et al. 2023). However, in youth with AN, there is currently no evidence to assume that psychotropic medications, including antipsychotics, lead to more weight gain (Deutsche Gesellschaft für Psychosomatische Medizin und Ärztliche Psychotherapie (DGPM) 2019; Frank et al. 2023; Frank and Shott 2016; Kishi et al. 2012). Furthermore, it should be taken into account that only small effect sizes were observed in the analysis of average weekly weight change. As described above, this finding underscores the assumption that weight gain in patients with AN is multifactorial (e.g., therapy adherence, metabolism, motivation, comorbidities, intensity of care) and largely standardised by treatments protocols (e.g., fixed nutrition plans; Deutsche Gesellschaft für Psychosomatische Medizin und Ärztliche Psychotherapie (DGPM) 2019), which limits the explanatory power of single factors.

Likewise, longer duration of inpatient treatment was significantly associated with a lower average weekly weight gain. It is not surprizing that a longer duration of inpatient treatment is negatively associated with weekly weight gain. In contrast to our study, however, longer duration of inpatient treatment was associated with a significantly greater change in BMI in previous studies conducted by Collin et al. (2010; *n* = 90 inpatients; 25.4 ± 6.8 years) and Schlegl et al. (2016; *n* = 238 female inpatients; 15.7 ± 1.1 years). These differences may be explained by different health care systems and the inclusion of different age groups. Children and adolescents are often referred to the clinic on the advice of primary care physicians and parents, often resulting in low levels of intrinsic motivation (Deutsche Gesellschaft für Psychosomatische Medizin und Ärztliche Psychotherapie (DGPM) 2019).

Our analyses showed that patients with AN+DSD were significantly more likely to report childhood emotional abuse compared to ANnoComorbidity patients. To the best of our knowledge, no study has examined the association between emotional abuse in childhood and depression in adolescents with AN. However, a correlation between emotional abuse and the risk of developing depression in adolescence is known (Courtney et al. 2008; Li et al. 2022). This is consistent with the “attachment theory”, which indicates, that an insecure parent‐child relationship has a negative impact on the development of depression in adolescents (Bowlby 1979; Li et al. 2022). Insecurely attached children have a lower self‐esteem and are less able to cope with stress and negative feelings. These are, in turn, risk factors for developing depressive disorders (Grawe 2017). At the same time, more severe AN and less weight gain have also been associated with suicidality and non‐suicidal self‐injurious behaviours in youth with AN (Arnold et al. 2022; Arnold et al. 2023), being yet another marker of AN illness severity and being also related to DSDs (Liu et al. 2022).

### 4.2 Limitations and Future Research

Results of this study need to be interpreted in the context of its limitations. First, this is a retrospective chart review that relies on data description and data entry into the basic data capture system by clinicians. Second, this study spans the years of 1994–2015, which introduces heterogeneity in terms of diagnostic and treatment procedures. Third, the duration of illness was asked retrospectively, introducing a recall bias as a methodological problem (Neubauer et al. 2014). Fourth, while many studies use the DSM‐III, ‐IV or ‐5 for making a diagnosis, patients in our study received a diagnosis of AN, atypical AN and of DSDs according to ICD‐10 (Dilling and Freyberger 2019). Due to this methodological difference, interpretability and comparison to studies conducted at other times or using other diagnostic systems may be limited. Fifth, the nature of data collection also makes it difficult to track which mental disorder (AN or DSD) came first in patients with AN+DSD. Sixth, the analyses did not consider the severity and type of depressive disorder, as rating scales of depressive psychopathology were not used. Seventh, we did not have sufficient information to determine improvement or resolution of DSD at discharge, which could have influenced the results. Future studies should measure depression in detail and over time. Eighth, other potentially relevant variables beyond DSD, such as different comorbidities, personality traits and family situation were not examined in detail. Ninth, we did not conduct structural equation modelling (SEM), as this was not feasible with the available data, since anthropometric parameters were only documented at admission and at discharge. Trajectory models require at least three repeated measurements per person to estimate changes in individuals over time (Curran and Hussong 2003; Nguena Nguefack et al. 2020). The restriction to two assessment time points limited our ability to assess potential indirect effects and complex relationships between influencing factors and weight change. Future studies should apply SEM or comparable mediation and path analyses to clarify underlying mechanisms. Finally, it is important to note that weight rehabilitation is not equivalent to the recovery of patients with AN (Solmi et al. 2024; Steinhausen 2002). Therefore, future studies should consider the therapeutic success of patients via different dimensions to evaluate eating behaviour, body satisfaction and well‐being (Herpertz‐Dahlmann et al. 2018) and also follow patients beyond discharge to track long‐term outcome of both AN and DSD as well as of their interrelationship. Nevertheless, despite these limitations, this study is one of the very few that investigated the effect of DSD in hospitalised youth with AN regarding weight gain and length of inpatient stay in a relatively large sample of well characterised children and adolescents hospitalised for the treatment of AN, taking advantage of a systematically implemented basic clinical documentation system.

In summary, the results of this study indicate that in youth with AN, DSD is a negative prognostic factor for the speed of weight gain during inpatient treatment and need for prolonged hospitalisation. Future studies should investigate what factors are responsible for and/or could remediate the differences in inpatient treatment outcomes (e.g., biological processes, psychotherapy, psychotropic medication treatment). Along those lines, it would be important to understand, whether the two patient groups benefit differently from the administered treatment programs or if the results indicate differences in eating behaviour or biological processes (Eskild‐Jensen et al. 2020). Further studies should aim to replicate these results and consider further variables, such as personality traits, environmental conditions and psychiatric disorders within the family, including the effects of past traumatic experiences, which recently have been replicated as a transdiagnostic factor for poor illness outcomes across different mental disorders (Solmi et al. 2023).

## 5 Conclusion

This is the first study investigating the extent and correlates of inpatient (average weekly) weight gain in youth with AN in relationship to comorbid DSD. Results indicate that patients with AN+DSD display lower average weekly weight gain during the inpatient stay compared to patients with ANnoComorbidity. Research approaches accounting for influencing variables, such as other comorbidities, personality traits and severity of illness, and following patients beyond hospital discharge are needed to disentangle the effects and interactions between AN and DSDs regarding the presentation and longitudinal outcome as well as most appropriate treatment approaches.

## Author Contributions

**Antonia Wiese:** conceptualization, data entry, data curation, formal analysis, methodology, software, visualization, writing ‐ original draft preparation. **Sabine Arnold:** data entry, data curation, writing ‐ review and editing. **Sarah Zaid:** data entry. **Antonia E. Müller:** data entry. **Christoph U. Correll:** conceptualization, resources, supervision, writing ‐ review and editing. **Charlotte Jaite:** conceptualization, project administration, supervision, writing ‐ review and editing.

## Funding

All authors were employed at the department of Child and Adolescent Psychiatry, Psychosomatic Medicine and Psychotherapy, Charité – Universitätsmedizin Berlin. Thereby, the study was financed by the Charité – Universitätsmedizin Berlin. The authors received no financial support specifically for this project, the authorship, or the publication of this article.

## Ethics Statement

The local Ethics Committee of the Charité – Universitätsmedizin Berlin, Germany, approved the retrospective chart review of routinely assessed clinical parameters (approval no. EA2/112/19).

## Consent

For this type of retrospective chart review based on routinely assessed clinical parameters patients formal consent was not required from the local Ethics Committee of the Charité – Universitätsmedizin Berlin, Germany.

## Conflicts of Interest

The authors Antonia Wiese, Sabine Arnold, Sarah Zaid, Antonia Müller, and Charlotte Jaite declare no conflict of interest. Christoph U. Correll has been a consultant and/or advisor to or has received honoraria from: AbbVie, Acadia, Adock Ingram, Alkermes, Allergan, Angelini, Aristo, Biogen, Boehringer‐Ingelheim, Bristol‐Meyers Squibb, Cardio Diagnostics, Cerevel, CNX Therapeutics, Compass Pathways, Darnitsa, Delpor, Denovo, Gedeon Richter, Hikma, Holmusk, IntraCellular Therapies, Jamjoom Pharma, Janssen/J&J, Karuna, LB Pharma, Lundbeck, MedAvante‐ProPhase, MedInCell, Merck, Mindpax, Mitsubishi Tanabe Pharma, Mylan, Neurocrine, Neurelis, Newron, Noven, Novo Nordisk, Otsuka, Pharmabrain, PPD Biotech, Recordati, Relmada, Reviva, Rovi, Sage, Seqirus, SK Life Science, Sumitomo Pharma America, Sunovion, Sun Pharma, Supernus, Tabuk, Takeda, Teva, Tolmar, Vertex, Viatris and Xenon. He provided expert testimony for Janssen and Otsuka. He served on a Data Safety Monitoring Board for Compass Pathways, Denovo, Lundbeck, Relmada, Reviva, Rovi, Supernus, and Teva. He has received grant support from Janssen and Takeda. He received royalties from UpToDate and is also a stock option holder of Cardio Diagnostics, Kuleon Biosciences, LB Pharma, Mindpax, Quantic and Terran.

## Supporting information


Supporting Information S1

## Data Availability

The data supporting the findings of this study are available on reasonable request from the corresponding author. The data are not publicly available due to privacy and ethical restrictions, due to the very high data protection of the data of children and adolescents in psychiatric institutions in Germany.
