# Supplementary material for: Comorbid Depression in Hospitalised Youth With Anorexia Nervosa: Characteristics, Correlates, and Impact on Weight Gain and Treatment Duration
Source: Eur Eat Disord Rev. 2026 May 27;34(5):1419–32. doi: 10.1002/erv.70130 (PMC13432646; doi:10.1002/erv.70130)
Supplement: Supplementary file 1 — Supporting Information S1 [file ERV-34-1419-s001.docx]

**Appendix 1: Overview of variables included in the regression model: analysis of the impact of clinical characteristics on average weekly inpatient weight change**

| **Which variables were significantly correlated with average weekly weight change (kg) in clinical analyses?** | **Was the significant variable included in the Multi variable stepwise backward elimination regression analysis?** |
| --- | --- |
| Affective disorders (ICD-10) | Included |
| Age at onset, years | Included |
| Age at admission, years | To avoid multicollinearity, the variable *age at admission in years* was excluded, as it is very similar to the variable *age at onset in years*, with a correlation coefficient of *r*=0.90 and *VIF*=7.42. |
| Year of admission | Included |
| Emotional childhood abuse | Included |
| Weight at hospital admission, BMI z-score | Included |
| Duration of inpatient treatment, days | To avoid multicollinearity, the *variable duration of treatment in days* was excluded, as it was used to calculate the dependent variable. |
| Weight at hospital discharge | The variable *weight at hospital discharge* was excluded because it occurs after average weekly weight gain and is therefore a result of this development rather than it’s cause. |
| Psychotropic medications, at least one | To avoid multicollinearity, the *variable at least one psychotropic medications* (*r*=0.99 and *VIF*=9.34) was excluded since this variable relied on the same data as the variables *treatment with Antidepressants and Antipsychotics.* |
| Psychotropic medications, Average number | To avoid multicollinearity, the variable *average number of psychotropic medications* (*r*=0.99 and *VIF*=10.13) was excluded since this variable relied on the same data as the variables *treatment with Antidepressants and Antipsychotics.* |
| Treatment with Antipsychotics | Included |
| Treatment with Antidepressants | Included |

**Appendix 2: Overview of variables included in the regression model: analysis of the impact of clinical characteristics on duration of inpatient treatment**

| **Which variables were significantly correlated with duration of inpatient treatment in clinical analyses?** | **Was the significant variable included in the Multi variable stepwise backward elimination regression analysis?** |
| --- | --- |
| Affective disorders (ICD-10) | Included |
| Weight loss in percentage, weight at admission compared to premorbid body weight | Included |
| Weight at hospital admission, BMI z-score | Included |
| Average weekly weight change, kg | To avoid multicollinearity, the variable average *weekly weight change in kg* was excluded, the variable *duration of inpatient treatment* was used to calculate this variable. |
| Psychotropic medications, at least one | To avoid multicollinearity, the *variable at least one psychotropic medications* (*r*=0.99 and *VIF*=9.32) was excluded since this variable relied on the same data as the variables *treatment with Antidepressants and Antipsychotics.* |
| Psychotropic medications, Average number | To avoid multicollinearity, the variable average number of psychotropic medications (*r*=0.99 and *VIF*=9.59) was excluded since this variable relied on the same data as the variables *treatment with Antidepressants and Antipsychotics.* |
| Treatment with Antipsychotics | Included |
| Treatment with Antidepressants | Included |
